# Raw Cotton Dressing for Wounds

**Published:** 1875-03-15

**Authors:** William H. Van Buren


### RAW-COTTON DRESSING FOR WOUNDS (PANSEMENTS A L'OUATE).

BY PROF. WILLIAM H. VAN BUREN, M.D.

*From New York Medical Journal, March,* 1875

I CANNOT leave this subject without calling your attention to a novel mode of dressing wounds with raw cotton, which has lately attracted much attention in Paris. I have already spoken of raw cotton as an American remedy in burns, but great advantages are now claimed for it as an application for recent wounds; it is said to protect them from the contact of the poisonous particles which are assumed as everywhere floating in the air as dust. These poisonous particles, in the present state of our knowledge, are supposed to be bacteria, or the spores of some analogous organism, capable of getting into the blood through open wounds, and acting as a ferment, or, at least, of being carriers of poison. So-called zymotic diseases, such as pyæmia and erysipelas, which interfere so often with the successful treatment of open wounds, especially in hospitals, are generally believed to owe their origin to this cause. Now, Pasteur, who discovered that putrefactive fermentation was due to the presence and growth of vegetable organisms, and Tyndall, who has experimented largely upon atmospheric dust, had both proved that raw cotton was the most effective filter by which the air can be deprived of these floating particles; that, when forced to pass through its meshes, air is rendered pure, as far as these poisonous particles are concerned. During the winter of 1870–71, when the hospitals of Paris were crowded with wounded soldiers and Communists, accumulated through the coincidence of the Prussian siege, the civil war, and famine, and when pyæmia was so universally prevalent that an amputation was almost of necessity fatal, this assertion of Pasteur concerning the filtering power of raw cotton occurred to one of the surgeons of the St.-Louis Hospital, Alphonse Guerin, as a possible source of advantage. He forthwith tried it as a dressing on several patients, binding it upon their wounds in liberal quantity, and keeping it accurately applied by firm compression of bandages. To his surprise and delight he found that the chill, by which the advent of the fatal complication is always heralded, did not occur, and his patients went on to get well. Encouraged by this experiment, he repeated it with equal success; those dressed with raw cotton were found to do well, while others in the same ward died of the prevailing endemic. The result was so remarkable that surgeons came from other hospitals to St.-Louis to witness the rare sight of patients recovering after amputation, and themselves adopted this mode of dressing wounds. Shortly, the use of raw cotton was systematized as a surgical dressing, and it has since been very generally employed. Desiring to learn the estimation in which it is now held, I sought information from my colleague, Dr. L. A. Stimson, who is at present in Paris, and I find that, although the prevalence of pyæmia has of course very much diminished in the hospitals, the raw-cotton dressing is still regarded favorably. It is claimed by Dr. Guerin, in recent communications to the French Academy (March 23 and May 18, 1874), that, in accordance with Pasteur's assertion, when properly applied, it effectually excludes a recent wound from contact with all poisonous germs—*without entirely excluding air.* He has found pus, in contact with a wound which had not been disturbed for thirty days, perfectly fresh in appearance and free from odor; it was changed in its anatomical and chemical characteristics, contained no traces of pus-cells, was converted, in short, into an oily emulsion containing large crystals of the fatty acids—showing that oxygen had not been excluded—but it presented no evidences whatever of putrefaction. In his earlier experience he once removed the dressing from two cases, which were doing perfectly well at the end of a week, and within three days both patients had chills, followed by fatal pyæmia. This led him to adopt the plan of applying the cotton-dressing with great care and precision at first, so that its renewal should not be necessitated until the establishment of a barrier of granulations, or the final healing of the wound. If the discharges saturated the cotton and made their appearance upon the surface of the dressing, additional layers of cotton wadding were applied over the old dressing without regarding bulk, and, over these, bandages put on with the greatest possible amount of compressing force; for it is found that where the cotton is applied in sufficient quantity, and of good quality, its elasticity renders the compression equable, and that, so far from being a source of danger, the elastic compression is advantageous. After a circular amputation of the thigh, an assistant steadies the stump, while another pulls apart the edges of the divided integument, and the surgeon proceeds to fill the cavity thus presented to him with small masses of cotton torn from the sheet of wadding, small at first and applied accurately to every part of the cut surface, then larger masses as it becomes filled, and then layers of the wadding are applied over and around the stump and upon the hip and pelvis, and over all a spica-bandage put on with great care, and as much compressing force as possible. No air must come in contact with the wound that has not filtered through the thick mass of cotton. Moreover, this cotton must be of good quality, fresh from the manufactory, and it must not have been exposed to the air of the hospital. Under favorable circumstances he has found it the best plan to leave this dressing in place about two weeks, when the granulating surfaces are usually found ready for approximation for final union, but he never renews a dressing in the foul air of a ward. Tarlatan and collodion straps are preferred to strips of plaster, as more transparent. He *has* obtained *primary* union. Guerin believes, with Lister and Tyndall, that while gases can be introduced into the blood through the lungs, poisonous particles like germs become entangled in the air-passages, and do not as a rule reach the blood by this route. The experiments and deductions bearing on this point are full of interest to the student of surgery, but the point is not as yet definitely settled; for Tyndall himself, in detailing the advantages of using a "cotton-wool respirator," remarks that "it is exceedingly probable that the germs which lodge in the air-passages, or find their way into the stomach with its absorbent system, are those which sow in the body epidemic disease." He adds: "If this be so, then disease can be warded off by carefully-prepared filters of cotton wool" (*vide* "Dust and Disease," in "Fragments of Science," etc., London, 1871, p. 321, *et seq.*).

It is a curious fact that Lister, the great advocate of the germ theory of disease, and of carbolic acid as a germ-killer, at one time was actually using raw cotton as a dressing for wounds, on account of its value as a filter, antecedent to its adoption by Guerin, but he afterward abandoned it in favor of his "antiseptic gauze" (*Pansements a l'Ouate*," par Raoul Hervey, *Archives Generales de Medecine*, December, 1871, *et seq.*). Guerin's advocates say that the Edinburgh surgeon was so pre-occupied and absorbed in finding out the best modes of using carbolic acid for the killing of germs, that he failed to recognize the probably greater value of the raw cotton as a filter in the dressing of wounds. It is one of the features of Guerin's mode of dressing, to wash a wound thoroughly with some germ-killing liquid, before applying the cotton; but he seems to attach as much value to weak camphorated spirits as to a solution of carbolic acid, using them indifferently.

In conclusion, it is claimed for this new method of dressing wounds, that besides protection from poisonous germs, it prevents inflammation, by its uniform elastic compression, the equability of temperature, the protection from external injury, and partial immobility which it confers; that it promotes the development and growth of granulations, saves the pain and trouble of frequent dressings, and facilitates the transportation of wounded men. It thus favors the scattering of the wounded after a battle, and for this reason, as well as the simplicity of the materials required, and the ease with which they can be applied, it commends itself to the military surgeon. In civil practice it promises to be useful, principally in the large hospitals of great cities, where pyæmia and erysipelas are always liable to become endemic, in preventing poisoning of open wounds by these diseases, and also by thus enabling surgeons to save limbs which might otherwise require amputation.

---

"Shadows from the Walls of Death."—Facts and Inferences prefacing a book of Specimens of Arsenical Wall Papers, gathered by R. C. Kedzie, M.D., Member of the State Board of Health of Michigan.—The above is the rather sensational title of an interesting and instructive paper upon an important subject. The danger attendant upon the use of paper hangings colored green with arsenical pigments (Scheele's green and Sweinfurt green—arsenite and aceto-arsenite of copper) is not so well known, either to the profession or to the public, as it ought to be. The State Board of Health of Massachusetts sounded the alarm, in 1872, by the publication in its annual report of an able paper "On the evil effects of the use of Arsenic in certain green colors," by Dr. F. W. Draper. The State Board of Health of Michigan now utters a similar warning, through this paper of Dr. Kedzie, and the accompanying book of samples of poisonous wall-paper.

"Perhaps," says the author, "we could not devise a more effectual way to contaminate the air of our houses with a small amount of arsenical dust than by the use of wall-paper colored with arsenical preparations. The large amount of surface exposed, the feeble adhesive power of the size by which the pigments are fixed, the frequent alternations of heat and cold, moisture and dryness, by which the adhesiveness of the size is still more diminished, the currents of air always circulating in a warm room, mechanical displacements by sweeping, dusting, etc., all combine to dislodge the pigments from their position on the paper, and to scatter them in the form of a fine dust in the room, and this dust may be many hours or even days in settling. * * * This suspended dust is swept along with the air in inhalation, and is lodged upon the mucous surface lining the nasal cavities, the windpipe and its ramifications. * * * This may explain the frequent occurrence of catarrh and bronchitis in those persons who occupy rooms prepared with arsenical wall-paper."

Dyspepsia, neuralgia, pains in the bones and joints simulating chronic rheumatism, headache, general debility, etc., are also enumerated as symptoms which often attend this insidious form of poisoning; their occurrence being readily explained by the absorption of the poison. Want of space, however, prevents our going into a full consideration of the subject, and we content ourselves with the citation of two or three of the cases which are reported by Dr. Kedzie as having occurred under his own observation, or within his knowledge.

1. Dr. J. H. B. and his two boys. The doctor's bed-room was papered with wall-paper of a grayish color, toned with green, and had a few bright green flowers or leaves. His boys occupied a bed-room next to his, and the door between the rooms was open at all times. The Doctor was troubled with severe pains in the bones, symptoms of chronic rheumatism, and constant cough. The boys became affected with pains and rheumatic soreness. Suspicion was aroused that the wall-paper might be the cause of this illness, and the paper was analyzed, when it was found to contain 5.47 grains of arsenic to the square foot, or six ounces of arsenic on the walls of a single room. * * * The paper was at once removed, and the Doctor and his boys entirely recovered.

2. The children of the Hon. L. D. W. Emma, aged nine, occupied a bedroom, the walls of which were covered with paper of a greenish stone color, with bright bands of green. Soon after occupying the room, she exhibited the following symptoms: Lameness, resembling rheumatism, darting pains in various parts of the body, languor in the morning, feverishness, pains in the head and frontal sinuses, sores in various parts of the body, "faint spells," and great loss of flesh. The best medical advice that could be procured was obtained, but no essential improvement followed. Whenever she left home for a time her health improved, but she relapsed into her former condition on returning home. The paper on the wall was analyzed, and found to contain 4.87 grains of arsenic to the square foot. Emma was removed from the room and entirely recovered her health.

3. Last fall, the son, Herbert, was allowed to occupy the same room. In a short time all the distressing symptoms which had afflicted Emma were developed in Herbert. He was removed from the room and entirely recovered.

4. Mr. H. had his house papered with wall-paper which contained a considerable quantity of green. Soon after Mrs. H. and all the children passed into a condition of continued ill-health. The time of the commencement of this ill-health was so nearly identical in all the cases, and the symptoms were so similar, that Mr. H. was convinced that there was some common cause operating on his family to cause this mysterious sickness, but he was unable to find anything in the condition of his home or its surroundings which would explain it. While examining the report of the State Board of Health, his attention was called to poisonous wall-paper as a possible cause of ill health. The wall-paper in his rooms was analyzed, and found to contain 1.88 grains of arsenic to the square foot. It was at once removed, and Mrs. H. and the children recovered their usual good health.

These cases require no comment. But as *all green wall-papers do not contain arsenic*, it is well to menttion the ready method of distinguishing those that do. "The green arsenical colors are readily soluble in ammonia water. If a little ammonia water poured on the paper discharges the green color, or produces such a change in the color as indicates the removal of green, the paper should be rejected, as it probably contains arsenic. To identify the presence of arsenic in any paper, wet the paper with ammonia water, pour off this water on a clean piece of glass, and drop into it a crystal of nitrate of silver, or a small piece of lunar caustic. If a yellow precipitate forms around the crystal, it indicates the presence of arsenic." L. S. J.—*Va. Med. Monthly.*

---

Is there Danger of "Driving in" a Skin Disease?—The popular opinion says there is. We have heard the venerable Prof. Geo. B. Wood sanction this opinion. Dr. McCall Anderson, in his late book on Skin Diseases, denies it, and scouts it. His reviewer, in the *Medical Press and Circular*, says:

"Eczema, known to have a constitutional origin, ought not to be suddenly cured. That eczema very often relieves the system by its appearance does not admit a doubt. In the young its sudden suppression has been followed by water on the brain, and here the disease had been confined to the head; and in after-life, when the gouty poison is so often mixed up with the eczema, we have known bad effects at once follow the disappearance of the rash. We have seen pemphigus make its appearance, and confined exclusively to the parts where the eczema had been. Violent cramps, too, are not uncommon under similar circumstances; and we have seen most obstinate cough and some forms of dyspepsia also come on. Every one, too, must have known bad effects follow the drying up of what seemed at the moment a very trivial discharge. This subject might be pursued very much further, but such is not called for here; and we would only add that on this point of the drying up or curing such a disease as eczema, too much caution cannot be exercised."

Here is an open question, and a very practical one, which should be discussed.—*Phil. Med. Reporter.*

Tannate of Quinine in Chronic Albuminuria.—Bouchardat (*L'Abeille Med.*, July 6) says: "I am in the habit of employing the sulphate of quinine in chronic albuminuria according to the method of Dr. Devouves, and occasionally with unhoped-for success. The dose I employ is eight grains in a cup of strong coffee three times a day. This is continued for six days, and at the end of that time scammony or some similar purgative is administered. After one or two days of rest, the patient is again placed on the use of quinine, and I have frequently continued this treatment for more than a year. The food of course should, during this treatment, be highly nutritious. Lately I have been substituting the tannate of quinine for the sulphate, in doses of ten to twenty grains, three times in twenty-four hours, given in a similar manner. The digestive apparatus supports the tannate better than the sulphate."—*Medical Times.*

---

Treatment of Whooping-Cough.—Wild claims that he can cure every case of whooping-cough within eight days by the following treatment: The patient is not to leave the room, and at every access of coughing is to hold before his mouth a small piece of cloth folded several times, and wet with a teaspoonful of the following solution: Ether, 60 parts; chloroform, 30 parts; turpentine, 1 part.—*Deutsches Archiv. f. Klin. Med. Allg. Wien. Med. Ztg.*, 45, 1874.

---

Formulæ for the Troublesome Cough of Phthisis:—

℞. Potassii bromidi,

Potassæ chloratis, } aa ʒ iss.

Ammoniæ muriatis,

Syrup. tolutani ℥ iv. M.

Tablespoonful every two or three hours.

℞. Tincturæ opii camphoratæ, ℥ i.;

" belladonnæ, ʒ i.;

" hyoscyami, ʒ ij.;

Spiritus Lavendulæ comp., ℥ i. M.

Ten drops on a lump of loaf sugar every hour until cough is relieved.—*Charity Hospital, New York.*

ON A RATIONAL TREATMENT OF PULMONARY PHTHISIS.—In a note presented to the Paris Academy, M. Pietra Santa upholds the doctrine of curability of pulmonary phthisis, after combating the German theory of cellular proliferation, and the fatalism of Broussais' school. For him, pulmonary phthisis is essentially a general and constitutional affection, a profound alteration of the acts of nutrition, a malady of the blood. Therefore, there is no panacea for a malady (symptomated by enfeebled vitality), of which the several phases of evolution form as many distinct morbid entities. There can be no antidote for a morbid diathesis, pre-existent to local anatomical lesions which characterize the affection. The unique specific for pulmonary phthisis is an intelligent and rational association of that collection of medicaments of which experience and chemical observation have made known the efficacy. The matter may be included in these precepts:

1. To call to aid, during all periods of the malady, all hygienic resources —moral and hygienic treatment, pure and renewed air, tonic alimentary regimen, moderate exercise, lactic diet.

2. To utilize mineral waters—sulphuretted, arsenical, chlorides.

3. To call to assistance the salutary effects of change of place, sojourn in temperate climates in winter, in mountainous countries during summer.

4. To neutralize the morbid ferments that engender in the organism purulent absorption, and lead to establishment of tuberculous matter. This is effected by the administration of hyposulphites and of alkaline and terrous sulphates.

5. Never to neglect the numerous general therapeutic agents, when these tend to combat the complications inseparable from each period of the malady.—*London Med. Record*, Dec. 20, 1874.

---

A HEALTHY YEAR.—It appears from the address of the President of the American Public Health Association that the present has been an exceptionally healthy year. This is shown by the reports now coming in of the various countries of the civilized world. Not only has there not been a movement of the great epidemics, but the general death-rate has been much less than during the preceding year.

---

A HINT IN GIVING IODIDE OF POTASSIUM.—A useful hint is revived in the *British Medical Journal* by Mr. Joseph P. McSweeney. He says: "Sir James Paget was the first to call the attention of the medical profession to the following interesting fact —namely, that carbonate of ammonia greatly increases the therapeutic action of iodide of potassium. I have had extensive experience in the treatment of syphilis, and have tried it with the best results, and find that five grains of potassium, combined with three grains of carbonate of ammonia, are equal to eight grains of the potassium salt administered in the ordinary way."

---

THE French Academy of Sciences recently made its awards of prizes for the years 1872 and 1873, distributing a total amount of over $11,000, gold, among twenty or more successful competitors, in sums of from $100 to $2,000.

With the exception of three small prizes in the department of botany, the prizes given were for investigations and discoveries in medicine and surgery; while in a few instances, appropriations appear to have been made for reimbursement of expenses of competitors who failed to obtain prizes.

At a meeting of the Academy in January of the present year, the announcement was made of the receipt from the Minister of the Interior, of a sum of $800, half of which is to be awarded to the best treatise upon the hygiene of infant children; the remainder to be expended in the publication of the successful work, and in encouragement.

THE INFLUENCE OF OZONE.—As heretofore remarked in this journal, the influence of ozone on health is esteemed of less and less magnitude. In the last Health Report of the British Navy is a paper on the subject by Staff-surgeon J. Mulvany. In conclusion he expresses an opinion that no disease can be clearly traced either to the superabundance or scarcity of ozone. Also that its most marked influence appears to be exercised over the generative organs; and as it tends to increase the chances of fecundation, the probability is that it imparts to the primary elements of life an accession of developmental force, which counteracts the transmission by the parents of that impaired bodily vigor which is always induced by the condition of heat, humidity, and clouds, etc., under which the manifestations of ozone are most liberal. And in this way ozone may be looked on as an important agent in the prevention of the degeneration of the human race. This is certainly a curious, and if correct, a conclusion of no trifling value.—*Phil. Med. Reporter.*

---

KEEPING THE BED AFTER CONFINEMENT.—Dr. William Goodell, of Philadelphia, in his account of the arrangement of lying-in women in the Preston Retreat, writes as follows in regard to the common practice of keeping the bed:

Lying-in women are encouraged to get up for food when they feel so disposed, because there are, to my mind, strong objections to the rigorous maintenance of the recumbent posture. Labor is, in general, a strictly physiological process, and there can be no sound reason why it should be made to wear the livery of disease. Nature teaches this very plainly, for most women wish to get up long before their physicians are willing to let them. The fact of a woman's wishing to get up is, to me, a very good reason why she should get up. In the second place, few physicians will deny that nothing so relaxes the tone of muscular fibre as a close confinement in bed. In my experience, a woman ordinarily feels stronger on the fifth day than she does on the ninth, if rigorously kept under quilts and blankets. Once more: the upright position not only excites the womb to contract, but, by distributing the blood and equalizing the circulation, it actually lessens the amount of the lochia and shortens their duration. On the other hand, the dorsal decubitus keeps up a passive congestion of the womb as a whole, the engorgement of the greatly hypertrophied placental site, and a blood-stasis in the now thickened posterior wall—all important factors in hindering the process of involution. Again: uterine diseases are hardly known among those nations whose women early leave their beds. From passages in the writings of the classics, it is evident that among the ancient Greeks and Romans, those models of physical strength and beauty, the women arose, and even bathed in a running stream, very shortly after delivery—in some cases on the very day. Finally, what is sounder than all theory, a sufficiently long and well-sifted experience has proved to me that by such a treatment convalescence is rendered far more prompt and sure. At this result, very unexpected to the multiparous patients of the Retreat, they are constantly expressing their surprise.—*Pacific Med. and Surg. Journal.*

---

TOO GOOD TO BE TRUE.—M. Paraf has discovered a way of doing without rain, if necessary. He knew that the air is full of moisture, and he knew that chloride of calcium would attract and condense it, for cultural purposes. He has applied this chloride on sandhills and roadbeds, on grass, on all sorts of soils, successfully, and he has ascertained that it may be applied in such proportions as will produce the irrigation of land more cheaply and efficiently than by means of canals or other methods of securing artificial irrigation.—*Boston Journal of Chemistry.*

NEW ANTISEPTIC OINTMENT. — Prof. Lister, according to the *Student's Journal and Hospital Gazette*, is at present using, with great success, an ointment composed of paraffin, 2 parts; white wax, 1 part; sweet oil of almonds, 2 parts; and boracic acid (powdered), 1 part. — *Medical and Surgical Reporter.*

---

TREATMENT OF PILES AND PROLAPSUS OF THE RECTUM BY INJECTION INTO THE RECTUM OF EXTRACT OF ERGOT.—Dr. G. William Semple, of Hampton, Va., reports (*Virginia Med. Monthly*) the case of R. M. B., whom he operated upon for piles in 1868 with the ecraseur. "The disease began to return in eighteen months, and increased to such an extent, accompanied by prolapsus of the rectum, that for three years there had been a considerable loss of blood every day, and it had been necessary that he should lie down after each evacuation of the bowels to await the return of the prolapsed rectum, when, on the 8th of April last, I operated on him by the application of nitric acid. This, contrary to my usual experience, produced violent pain and irritation of the neck of the bladder, requiring for three days large and repeated doses of morphia.

"After healing of the slough, the piles still continued to bleed and to be painful, but the patient was unwilling to submit to further operative procedure. Having, at the same time under successful treatment, by ergot, a mammary tumor, and my attention having been called to the relief of varicose veins by the hypodermic injection of ergotin, and remembering that in several cases of imperfect involution of the uterus, in which I had successfully prescribed ergot in combination with sulphates of quinia, iron and strychnia, and extract of cannabis indica, in which piles coexisted, they also had been cured, I ordered the injection of ʒ ss. of the fluid extract of ergot, with ℥ ss. of water, into the rectum after each evacuation. Though the patient has been irregular in pursuing the treatment, he has since seldom had any discharge of blood or suffered any inconvenience, and now considers himself cured.

"On the same day (May 1) this prescription was made, B. H. L., convalescent from typho-malarial fever, who had suffered with piles and prolapsus of the rectum for fifteen years, daily losing blood, and being obliged to lie down after every evacuation to await the return of the prolapsed part before resuming his work, called on me for advice. Ordered the same treatment, which he pursued steadily. At the end of six weeks, having had but one discharge of blood, he felt himself well, saying he was a new man.

"This patient's spleen had been greatly enlarged for many years, and that, much to my surprise, was reduced to its normal size by the treatment.

"I have since prescribed the same treatment in three other cases of piles, all of which have been cured. One of the patients was a pregnant woman. No labor-pains were produced, or inconvenience felt."

---

DIGITALIS. — M. Gaunot, in the course of a paper published in the *Gazette Medicale de Paris*, on the action of digitalis, says: "When digitalis or digitalin is administered for some time to a man in full possession of sexual powers, these become gradually weakened, the propensities disappear, formation of the liquor seminis diminishes, and may at last cease altogether. The anaphrodisiac properties of the drug are the secret of its good effect in spermatorrhœa."

Dr. Little recommends the use of digitalis in febrile cases in which stimulants are either not well borne or are contra-indicated, as where renal affection is present. He has given half-drachm doses of the tincture every three or four hours in typhus, enteric and rheumatic fevers, with very favorable results. — *Edinburgh Medical Journal.*

PUERPERAL FEVER. — In the address in obstetrics, delivered at the last annual meeting of the British Medical Association,* Dr. J. Matthews Duncan stated that it was his belief, that nearly one in every one hundred women, delivered in Great Britain at or near the full time, died in parturition, or before the puerperal state and its effects had passed away. In addition to this startling mortality, parturition brings with it a vast amount of disease and suffering which does not end fatally. By far the largest number of deaths are due to puerperal pyæmia, a term which Dr. Duncan would substitute for the erroneous one of puerperal fever, since there is nothing essentially puerperal known in it, nor anything of the nature of a fever as that term is generally understood. All evidence brings the disease into the closest alliance or identity with surgical pyæmia. It is a disease always present ; but never has it been shown to possess an epidemic character. It varies in its ravages just as pneumonia does, but in a very different manner from what is found to be the case with cholera or scarlatina. The puerperal woman presents a most favorable nidus for the reception of morbific material in her contused and lacerated passages, and patients suffering from puerperal pyæmia, or any allied diseases, offer this morbific material in its most potent essence. No other well demonstrated communicability has ever been proven, as tending to show that this disease is either contagious or infectious. The same laws of pathology govern lying-in hospitals as govern all hospitals, and it is absurd to claim that any greater danger exists for patients in one than for those in the other. Beyond question, pyæmia is a septic disease, and puerperal pyæmia may be almost if not altogether prevented by the application to delivery of a practice based on antiseptic principles.

In a carefully prepared treatise on Erysipelas and Puerperal Fever, recently published, the author, Dr. Thomas C. Minor, gives in detail an account of both diseases as they prevailed sporadically in the United States during the year 1870, and also the history of a puerperal fever epidemic observed in the southwestern part of Ohio in the winter of 1872. The facts, as given by Dr. Minor, appear to establish the fact that erysipelas and puerperal fever seem to prevail together throughout the United States, and that any marked increase of one disease in any particular locality, is always followed by a corresponding increase of the other. Infants die of erysipelas shortly after or before their mothers die of puerperal disease.

M. Chauffard * considers that any disease which produces morbid discharges may give rise, in women recently confined, to puerperal fever. During his service at the Neckar Hospital, he has frequently remarked that the opening of an abscess, or the presence in the wards of purulent ophthalmia or erysipelas, is sure to be followed in a short time by puerperal troubles of greater or less severity.— *Boston Med. and Surg. Journal.*

---

ENLARGEMENT OF THE TONSILS AS A CAUSE OF NIGHTMARE.—J. Warrington Haward, F. R. C. S., relates some cases of nightmare occurring in children, resisting ordinary methods of treatment, and increasing in frequency and severity, which were at once permanently cured by the removal of a portion of the tonsils. As these glands were enlarged, he was led to believe that the obstruction to respiration and consequent cerebral congestion were sufficient to produce the disorder. Nightmare due to gastric irritation or dentition occurs, as a rule, only once in the night, while that due to enlarged tonsils often recurs several times in the same night, and is invariably observed to be aggravated by the child catching cold.— *British Medical Journal.*

* *Obstetrical Journal of Great Britain*, Sept., 1874.

* *Gazette Hebdomadaire*, October 16, 1874.

THE TENSILE STRENGTH OF THE FŒTUS.—Dr. J. Matthews Duncan reports* a series of experiments undertaken to ascertain the amount of force which can safely be applied in effecting the delivery of a child. The body of a new-born baby was passed through an aperture so cut in hard wood as to represent the brim of a contracted pelvis. Above the ankle, an apparatus was applied by which weights could be suspended. The amount of the weights used was to be gradually increased until the body of the child was dissevered. In order to bring the experiment into conditions analogous with those of labor, the weights were allowed to operate for only half a minute at a time. In each experiment the separation took place at the neck. The decapitating force varied from ninety-one to one hundred and forty-one pounds, giving, therefore, an average of about one hundred and twenty pounds.

The life of the child is, of course, compromised before the limit of the tensile strength of the fœtus is reached, since the destruction of the spinal column occurs under the application of a force considerably less than is required to produce decapitation.

In these experiments it was found that the cervical part of the vertebral column first gave way, allowing the dissevered vertebræ to become widely separated before actual decapitation took place. While one hundred and five pounds sufficed to separate the cervical vertebræ, an addition of fifteen pounds was required to decapitate. In all cases the vertebral column yielded with a jerk, followed by a marked elongation of the fœtal body. This fact is, therefore, one of great importance to the accoucheur if he wishes to avoid decapitation. It was further found that a single limb, so far as strengh was concerned, sufficed to effect decapitation by traction.

These experiments are of great value as regards the force that may safely be employed in podalic extraction; but it was found a far more difficult problem to ascertain the power that may safely be applied in extracting a child with the forceps. In this latter case, a far greater force can be exerted than in the former. It has been proven that it is possible to apply the forceps to a fœtal head at the brim of an actual pelvis, so as to defy the utmost efforts of the most powerful accoucheur. The fœtal head is firm enough to resist a dragging force far greater than can be used in podalic extraction.—*Boston Med. and Surg. Jour.*

* *British Medical Journal*, December 19, 1874.

---

IN cases of dropsy of the joints, especially that of the knee, Dr. Bergeret finds the continued application of bags of hot sand to answer better than any other kind of treatment. When the acute stage is passed, and whatever may be the cause of the dropsy, he wraps the joint in a thick layer of cotton-wool, and applies to this a sack containing two or three litres of fine and very hot sand. The dropsy disappears in a few days. The sand must be very hot, and the heat may be kept up by means of covering with a blanket. The sand must not be too thick in the bag, so that it may extend easily on the knee, and overhang the hydrarthrosis in every direction.—*Boston Medical and Surgical Journal.*

---

DIPHTHERIA.—Dr. J. Lewis Smith, (*N. Y. Medical Record*) says: The local treatment consists in the application to the fauces, every three hours, of the following, which has given me better satisfaction than any other medicine for local treatment which I have employed: ℞. Acidi carbolici, gtts. v.; liq. ferri subsulphat, ʒ ij.; glycerin, ℥ i. M. The brush was employed in preference to the probang, as it is less painful, and where there is a pseudo-membrane does not produce hæmorrhage by its detachment,

INCISION OF THE CERVIX UTERI.—Prof. R. Olshausen * divides dysmenorrhœa into three varieties, according as its origin may be considered obstructive, congestive, or ovarian. Incision of the cervix uteri is to be used only in that class of cases, small in number, where the dysmenorrhœa is dependent upon the small size of the external os. In cases where either the internal os or the cervical canal is found to be narrowed by some lesion of the lining mucous membrane, the cervix uteri should never be incised. In all cases where the sound enters the uterine cavity suddenly, as if passing some contracted spot, whether that contraction be owing to disease, or is the normal condition of things, an incision should be made. Wherever sterility is found to exist with mechanical dysmenorrhœa, there is always a pathological contraction of the external os. In those cases in which we are attempting the removal of sterility, the external os may be incised, even though it appear to be normal. It is a well-known fact that the dilatation of the os, as occurs in labor, is favorable to a subsequent conception, since many women, who have been sterile for a long time, gave birth to a number of children rapidly after the birth of the first child.

The best instrument to be used in performing incision is Sims' blunt-pointed bistoury. The blade should in all cases be passed as high up as it is desirable to make the incision, and the cut should be made from above downwards. The instrument should then be withdrawn, and the opposite side cut in the same manner. It is very important that the external os should be so deeply cut as to leave an open slit. The lips are then to be kept apart by tearing any adhesions which may form every twenty-four or forty-eight hours, and a solution of the perchloride of iron should be subsequently applied. Sponge tents should never be used, since they are liable to produce septicæmia. The operation should never be performed if there is any inflammatory process going on in the neighborhood of the uterus.

In those cases where there is anteflexion of the uterus, accompanied by sterility, a good result seldom follows the ordinary lateral incisions. In these cases, a wedge-shaped piece should be cut out of the anterior lip. The bilateral incision of the os uteri is recommended in all severe cases of uterine catarrh, since internal medication of the uterine cavity is then rendered more easy of application.

The operation of incision is one worthy of the most careful study, and requires the greatest care in the performance of it, as well as in the after-treatment of the patient.—*Boston Med. and Surg. Journal.*

* *Volkmann's Sammlung klin. Vortrage*, 67, 1874.

---

CARBOLIC ACID IN TAPEWORM.—Dr. S. A. Brown, U. S. N., writes to the New York *Medical Record:* In a recent issue of your journal I noticed a letter from Dr. Bills, of the army, recommending the use of carbolic acid in the treatment of tapeworm.

Having a case on hand at the time which had resisted the use of oil of turpentine and pepo for a month or six weeks, I immediately administered to him the following prescription: ℞. Acid carbolic cryst., gr. ij.; glycer. pulv. gr. iv. Ft. pil., No. 2. To be taken every three hours, beginning at 9 a. m. and continued until 9 p. m. This followed in the morning by jalap and rhubarb, ten grains each. On the first day a few joints were passed; on the second a great number, and on the third the head and narrow portion came away, with a great number of small sections, from one to three inches long. On the fourth day, treatment being kept up without further result, the medicine was discontinued. The only sensation created in the stomach by the administration of such large quantities of the drug was a slight burning, not sufficient to inconvenience the patient. I look upon this as a perfect cure, and therefore report the case to you.

HÆMORRHOIDS.—Dr. Wm. Colles, Dublin, lately injected twenty minims of tincture of perchloride of iron into each internal hæmorrhoidal tumor. No traces could be found some weeks afterward, by speculum, except nodules, of the size of a shrivelled currant. The case had resisted Dr. Houston's application of fuming nitric acid.—*British Medical Journal*, June 27, 1874, p. 849.

---

RESEMBLANCE OF TWINS. — Dr. Bird declares that, according to his own experience during thirty years, twins which are contained in the same membranes, and have a single placenta, resemble each other; while those which are contained in separate membranes, and have different placentæ, do not resemble each other more than other children of the same parents.—*La Tribune Medicale.*

---

ASPIRATION IN PLEURISY. — The question as to the use of the pneumatic aspirator in pleurisy has of late been discussed by Dr. Becker, of Munich. In cases where the operation is one of necessity for the preservation of life, there can, of course, be no question as to its propriety; but in most cases of pleurisy with sero-fibrinous effusion, Dr. Becker is decidedly opposed to aspiration of the fluid. Exen where the pleural cavity is so full that the heart is much displaced, as long as the respiration and circulation are not disturbed, he would leave the cure to nature. He contends that when the fluid has reached a certain amount, effusion ceases, and the current setting in an opposite direction towards the vessels, the cavity is gradually emptied, and contraction and adhesion occur in due order. Nature spontaneously limits the amount of effusion by compressing the root of the collapsed lung, and thus so far arresting the circulation in the vessels of supply. If the physician interferes with this normal course of affairs, and removes the fluid before the pressure in the pleural cavity has reached a certain height, he will simply restore the circulation in the pulmonary vessels, re-establish the conditions of effusion, bring the rough surfaces of the pleura into frictional contact, and have robbed the system of so much precious fluid.

Dr. Becker considers the circumstances even more serious when the collapsed lung is adherent. Should aspiration then be performed, the fluid speedily refuses to flow, the tube collapses, and air forces its way around the needle into the chest. Worse still, the lung expanding unequally may undergo alveolar dilatation; it becomes hyperæmic, hæmoptysis may occur at once, and bronchitis and pneumonia supervene. Less serious reasons for letting nature alone in sero-fibrinous pleurisy without urgent symptoms are the facts that the risk of sudden death from fatty heart, which is present in such cases, is not removed by operation; that marasmus is not relieved by it; and that fresh pleurisy often comes on, and the chances of empyæma increase with every tapping.—*Boston Med. and Surg. Jour.*

---

ETHER FOR TAPE-WORMS.—When the anæsthetic power of ether was first discovered, it was only proposed to use it on human beings to render surgical operations painless. Von Heyden, the merciful man who would not inflict pain on any living creature, employed it as long ago as 1830 for killing insects for his collection. Even worms are rendered dormant and helpless by its use. Prof. August Vegel now announces a new application of this anæsthesia for worms —its application to tape-worms. The ether is enclosed in a gelatin capsule and swallowed. The ether is vaporized in the stomach, and the worm stupefied, it being then easily removed by any of the usual remedies, against which when awake, the worm offers a strong resistance.

THE VALUE OF QUINIA IN ACCELERATING PARTURITION.—Dr. O. B. Stafford, of New Boston, Ill., reports (*N. Y. Med. Jour.*, Feb. 1875,) several cases illustrating the value of quinia in accelerating parturition. In his first case his patient had been in labor for twenty-four hours, but for two hours previous to his seeing her, had been without any pain whatever. Found a well-dilated bag of waters presenting properly, but everything pertaining to active labor positively quiet. He gave her eight grains of sulphate of quinia at one dose, and in twenty minutes strong expulsive labor-pains set in, and in one hour labor was terminated.

In another case the patient had been in labor about eighteen hours, but aside from presentation of bag of waters and dilatation of os uteri to the size of a shilling, no progress had been made. After waiting two hours, and finding that manipulation about the os failed to produce any uterine contraction, Dr. Stafford gave her about seven grains of quinia. In thirty minutes, patient complained of strong bearing-down pain. Uterine walls hardened, dilatation was completed, and in one hour and a half delivery was completed.

One very remarkable feature Dr. Stafford finds, presents itself in the administration of quinia in obstetrical cases. It does not produce that *spasmodic* uterine contraction, which in his hands at least, so frequently follows the giving of ergot; also an unusual freedom from flooding; the entire placental mass is dislodged with but little difficulty, the uterus contracts firmly, presenting that hard, woody feeling which is so pleasing to the careful obstetrician.

In all his cases, the mothers convalesced well. General nervous prostration did not ensue to so great an extent as we frequently observe. Lochial discharges were normal, and there was a marked freeness from malarial difficulties, puerperal fever, chills, etc., which are so prevalent in the Mississippi Valley.

EXTERNAL USE OF TURPENTINE IN THE TREATMENT OF TONSILITIS.—In the *Leavenworth Medical Herald* Dr. S. H. Roberts strongly recommends the use of turpentine externally in tonsilitis. He folds the flannel to four thicknesses, wrings it out in hot water, and pours oil turpentine over a spot the size of a silver dollar. The flannel is then applied over the sub-parotid region, and the fomentation continued as long as it can be borne. After removal a dry flannel is applied, and the same region rubbed with turpentine every two hours. This application is continued daily till resolution occurs. The doctor believes, from the evidence of his long experience, that thus applied early in the disease, the oil of turpentine has almost a specific effect in tonsilitis. That its action is not simply that of an irritant, he has proved by employing mustard, croton oil, tr. iodine, etc., in the same class of cases. They always failed to diminish the inflammation of tonsils, while the turpentine succeeded.—*Detroit Review of Med.*

A SIMPLE METHOD OF REDUCING THE DISLOCATION OF THE FOREARM BACKWARDS.—Dr. Alexander Murray writes to the *New York Medical Record* of July 1, 1874, that he has reduced five cases of the above-mentioned dislocation by the method to be described.

Supposing the dislocated arm to be the left. Dr. Murray takes his position at the outside of the dislocated arm, and places the palm of his right hand to the patient's left, dove-tailing his fingers between each of the patient's. In this way, a firm hold is secured for extension. He then places his elbow as a fulcrum and for counter-extension on the forearm in front and against the lower end of the humerus, and by a steady pressure downward and backward, and at the same time flexing the forearm toward the shoulder, in a few minutes the luxated bones slip into their natural places. Other dislocations of the elbow can be reduced by the same method.

